# Pro-inflammatory granzyme K contributes extracellularly to disease

**DOI:** 10.3389/fimmu.2025.1620670

**Published:** 2025-06-18

**Authors:** Christopher T. Turner

**Affiliations:** Future Industries Institute, University of South Australia, Adelaide, SA, Australia

**Keywords:** granzyme, inflammation, serine protease, CD8 lymphocytes +, cytotoxicity and immune system

## Abstract

Granzyme K (GzmK) is an immune-secreted serine protease typically expressed at low levels but elevated in response to tissue injury and disease. Known as an orphan granzyme due to limited scientific investigation, this tryptase is being redefined as having important roles in inflammation and disease pathogenesis. Multiple GzmK expressing CD8^+^ T cell subsets are being identified with augmented expression and important roles in disease. Traditionally recognized as a mediator of cytotoxic lymphocyte-mediated cell death, GzmK’s role is being recharacterized through multiple recently released studies focused on newly identified extracellular mechanisms of action. These studies identify GzmK to be inflammatory, being able to trigger pro-inflammatory cytokine release, enhance immune cell recruitment, exacerbate the immune response to bacterial infections, and activate complement. In multiple disease states, dysregulated GzmK expression and potential accumulation in the extracellular space directly contributes to impaired health outcomes, thereby suggesting downregulation may prevent disease severity. GzmK is therefore emerging as a therapeutic target, potentially valuable in sepsis, pulmonary disease, inflammatory skin disease, rheumatoid arthritis and even aging.

## 1 Introduction

### 1.1 Granzymes

Granule-secreted enzymes (granzymes) are a family of serine proteases identified to mediate cell death by natural killer cells and cytotoxic T lymphocytes (1–5). There are five human granzymes, comprising tryptases granzyme A (GzmA) and GzmK, aspartase GzmB, chymase GzmH, and metase GzmM. Despite sharing structural sequence homology and a conserved secondary structure (6, 7), granzymes exhibit distinct substrates and varied roles in both healthy tissues and pathologic ones in multiple disease modalities. GzmA and GzmB are the most extensively studied granzymes, while the others are less well elucidated, thus referred to as ‘orphan’ granzymes. In recent years, several emerging studies have focused on GzmK, revealing significant implications in various diseases and offering new insights into mechanisms of action. As a result, investigating GzmK has become an exciting area of active research.

### 1.2 Granzyme K

GZMK, the human GzmK gene (EC: 3.4.21) is located on chromosome 5.q11.2 and encodes a 264 amino acid protein. Also known as granzyme-3, fragmentin-3, or NK-tryptase-2, GzmK is synthesized in the rough endoplasmic reticulum as a zymogen precursor and then stored in granules, where it is associated with the proteoglycan serglycin. To become proteolytically active, the proteinase cathepsin C (also known as dipeptidyl peptidase I) performs NH_2_-terminal processing (8). As a highly cationic tryptase-like protease, GzmK cleaves after basic amino acids, preferentially after positions 6 and 9 but also after positions 7 and 8 (9). Since both GzmA and GzmK are tryptases, have closely related three-dimensional structures (9) and share some common substrates, GzmK was long considered a redundant enzyme to GzmA. GZMK is located near GZMA on chromosome 5, likely due to gene duplication. However, the idea that GzmK is merely redundant to GzmA is now rejected, as GzmK has unique substrates and functions that distinguish it from GzmA. GzmK and GzmA also shows wide structural variation around the active subsites (10).

### 1.3 Granzyme K expression is elevated or augmented in multiple disease states

GzmK detection in plasma and tissues is low in healthy conditions but becomes elevated in response to disease/tissue injury, specifically accumulating in regions of inflammation (**Table 1**). GzmK is elevated in bronchoalveolar lavage fluid from acute bronchopneumonia patients and allergic asthma subject’s post-allergen challenge, but not mild chronic obstructive pulmonary disease (11). Plasma GzmK is elevated in patients experiencing sepsis (12) and Dengue fever (13). In renal transplant patients with immunosuppressive therapy and suffering from cytomegalovirus infection, plasma GzmK is elevated and associated directly with the infection (13). GzmK is also transiently elevated in circulation following lipopolysaccharide (LPS) administration (14). GzmK is only released upon stimulation with *Pseudomonas aeruginosa*, but not *Escherichia coli BL21*, and *Neisseria meningitidis*, suggesting upregulation is pathogen specific. Tissue GzmK levels predict chronic rhinosinusitis-associated nasal polyp recurrence and asthma comorbidity (15). GzmK is also elevated in skin damaged by acute burn injury (16), and lesions in the inflammatory skin diseases, psoriasis (17) and atopic dermatitis (18).

**Table 1:** GzmK detection documented in disease.

Disease/injury	Tissue	Cell source	Species	Reference
Aging, intrinsic	PBMCs Spleen, peritoneal cavity, lungs, liver	CD8^+^ T cells CD8^+^ T cells	Human Mouse	Mogilenko et al., 2021 (19)
Alzheimer’s disease	PBMCs	CD8^+^ T cells	Human	Duan et al., 2023 (23)
Amyotrophic lateral sclerosis	CSF	CD8^+^ T cells	Human	Kim et al., 2024 (28)
Asthma	BALF BALF	CD8^+^ T cells, CD8^+^ T cells	Human Mouse	Bratke et al., 2008 (11) Lan et al., 2025 (15)
Atherosclerosis	Atherosclerotic plaques	CD8^+^ T cells	Mouse	Tyrell et al., 2023 (25)
Atopic dermatitis	Skin	mast cells, others?	Human, mouse	Turner et al., 2022 (18)
Burn (acute thermal injury)	Skin	Mast cells, M1 macrophages	Human, mouse	Turner et al., 2019 (16)
Cancer	Liver, adipose, Tumour	CD8^+^ T cells	Human	Duquette et al., 2023 (39)
Crohn’s disease	PBMCs	CD8^+^ T cells	Human	Lee et al., 2025 (60)
Chronic rhinosinusitis	Blood, nasal tissue	CD8^+^ T cells	Human	Guo et al., 2024 (61)
Psoriasis	Skin	Mast cells, others?	Human, mouse	Richardson et al., 2024 (17)
Rheumatoid arthritis	Synovial tissue, blood	CD8^+^ T cells	Human	Jonsson et al., 2022 (27)
Sjögren’s syndrome	Salivary glands	CD8^+^ T cells	Human	Xu et al., 2023 (26)
*Infection*				
Acute bronchopneumonia	BALF	CD8^+^ T cells	Human	Bratke et al., 2008 (11)
Dengue fever	plasma	NKT	Human	Bade et al., 2005 (13), Choi et al., 2024 (62)
HIV	PBMCs	GzmB^+^CD8^+^ T cells	Human	Zhao et al., 2024 (63)
Sepsis	Plasma Spleen	Undefined NK and NKT	Human	Rucevic et al., 2007 (12) Uranga-Murillo et al., 2021 (31)
viral infection	Plasma	Undefined		Rucevic et al., 2007 (12), Bade et al., 2005 (13)
viral pneumonia	BALF	Undefined	Human	Bratke et al., 2008 (11)
endotoxemia	Plasma	Undefined	Human	Wensink et al., 2016 (14)

PBMCs = Peripheral blood mononuclear cells, CSF = cerebrospinal fluid, BALF = bronchoalveolar lavage fluid.

Specific immune cell populations, and in particular T cells, have augmented GzmK expression in response to certain disease states, including rheumatoid arthritis, amyotrophic lateral sclerosis, and aging. A subset of GzmK^+^ exhausted memory T cells (Taa) has been identified to accumulate with age in the lung, liver, peritoneal cavity, and spleen (19). Separately, GzmK^+^ CD8^+^ T cells were found to be higher in the plasma of older adults (20). Humans seropositive for cytomegalovirus exhibit higher GzmK^+^ CD8^+^ T cells. GzmK^+^ NK cell frequency is inversely correlated with antibody titers pre-and post-influenza vaccination. GzmK^+^ T cells are increased in both cancer and inflammaging, including squamous cell carcinoma (21), melanoma (22), Alzheimer’s disease (23), and atherosclerosis (24, 25). In Sjögren’s syndrome patients, there is an increased proportion of CXCR6^+^GzmK^+^CD8^+^ T cells in the peripheral blood, with these displaying an activated phenotype (26). In rheumatoid arthritis, GzmK^+^ CD8 T cells are enriched, with these greater than 10% of all live cells in inflamed RA synovium (27). GzmK^+^ cytotoxic T cells were also found to be a major CD8^+^ T cell population in gut samples from Crohn’s disease patients and bronchoalveolar lavage fluid samples from COVID-19 patients, with these enriched in diseased tissue but also found in circulation (27). Finally, there is a higher proportion of CD8^+^GzmK^hi^ effector memory T cells in the cerebrospinal fluid of patients with amyotrophic lateral sclerosis (28).

### 1.4 Granzyme K contributes to disease

The development of a GzmK knockout (GzmK^-/-^) mouse (29) has allowed elucidation of the biological role of this protease in a variety of disease states. Comparing GzmK^-/-^ and GzmA^-/-^ mice has allowed confirmation that there is the lack of overlap between the functions of GzmK and GzmA. GzmK^-/-^ mice exposed to Chikungunya virus infection displayed reduced foot swelling, although this is less than observed in GzmA^-/-^ mice (30). Sepsis scores are also reduced in GzmK^-/-^ mice compared to WT mice, however, only GzmA^-/-^ mice have improved survival (31).

In acute burn injury, GzmK^-/-^ mice resolved inflammation faster, and improved wound closure, quality of healing, and scar strength compared to wild-type mice (16). Separately, in oxazolone-dermatitis (18) and imiquimod-psoriasis (17) models of inflammatory skin disease, severity is reduced in GzmK^-/-^ mice. In the dermatitis mice, GzmK^-/-^ mice display reduced scaling, erosions and erythema, with an associated improvement in angiogenesis and decreased microvascular damage. In the psoriasis mice, GzmK^-/-^ mice have reduced plaque formation, less erythema, and decreased epidermal thickening. Using a different GzmK^-/-^ mouse, this time with skin exposed to imiquimod to induce skin inflammation, there is decreased erythema, scaling and skin thickness (15). In mouse asthma models, GzmK knockdown or pharmacological inhibition decreased tissue pathology and restored lung function (15). GzmK^-/-^ mice display reduced arthritis severity and dermatitis with reduced complement activation.

## 2 Different schools of thought: intracellular versus extracellular roles for GzmK

### 2.1 Intracellular roles for granzyme K

Historically, all granzymes were believed to mediate cytotoxic lymphocyte-mediated cell death. Upon target cell engagement, granules release their granzyme payload into the immunological synapse. The pore-forming protein perforin is released in conjunction with these granzymes and facilitates granzymes entry into the target cells. Once internalized, granzymes induce cell death through caspase-independent or -dependent mechanisms (reviewed in (32)). The specific details related to these mechanisms remain unclear and have not been independently confirmed. As such, this remains an area of controversial area, especially the idea that GzmK contributes to cell killing, and has been questioned by several independent studies (33).

### 2.2 Extracellular roles for granzyme K

There are three main key indicators that GzmK is released from cells and into the extracellular space: 1/leakage from the immunological synapse, 2/secretion from non-cytotoxic and possibly non-immune cells, and 3/interaction with extracellular substrates. Notably, the GzmK^+^ CD8 T cells found to have a relatively increased expression in multiple disease states minimally express cytotoxic markers (27), suggesting extracellular roles may be especially important in disease.

#### 2.2.1 Leakage from immunological synapse

GzmK is expressed in diverse populations of cytotoxic cells, including CD8^+^ T cells (γδ T cells, mucosal-associated invariant T (MAIT) cells, a subset of non-MAIT CD8 T cells, CD8^+^GzmK^hi^ T cells, and CD45RO^+^CCR7^+^ and CD45RO^+^CCR7^-^ CD8 T cells) natural killer cells (CD56^bright^ and invariant NKT) and cytotoxic CD4^+^ T cells (27, 28, 34–36). Following target-cell engagement and granzyme release into the immunological synapse, only an estimated two thirds are internalized with the remainder dispersed into the extracellular milieu (37). Recently, CD8^+^ T cells were found to secrete GzmK in the absence of T cell receptor stimulation, supporting constitutive synthesis and secretion (38).

#### 2.2.2 Non-cytotoxic cells express and secrete GzmK

Multiple GzmK^+^ cells are non-cytotoxic, with these cell types secreting no perforin and/or unable to form immunological synapses (39). These cell types include macrophages (16), non-cytotoxic CD56^bright^ CD16^−^ natural killer cells (35), and mast cells (17, 18). In cultured macrophages, GzmK is constitutively secreted from M1 but not MØ or M2a macrophages (16). In dual GzmK/TBO stained mast cells, extracellular GzmK^+^ vesicles were also observed following degranulation (18).

#### 2.2.3 GzmK cleaves extracellular substrates

GzmK is potently inhibited in human plasma by the inter-alpha-inhibitor protein complex (IαIp), leading to speculation of the existence of extracellular GzmK substrates (11). Multiple extracellular substrates have now been identified within the extracellular matrix and on cell surface membranes. These include cleavage of Protease-Activated Receptors (PAR) (40), complement C2 and C4 (38), LPS (41), syndecan-1 (18) and decorin (18). The use of degradomics and other techniques will allow further identification of additional extracellular substrates.

In summation, the data now suggests extracellular GzmK as having an emerging role in disease pathogenesis, likely more so than GzmK-driven cell-mediated cytotoxicity. Fundamental to this idea is the need for a better understanding of the extent extracellular GzmK accumulates in diseased tissue, what kinds of tissues, and whether the amount of accumulation directly correlates to disease severity.

## 3 Current research gaps: does extracellular GzmK accumulate in diseased tissue?

In injured/inflamed tissues collected from a variety of diseases, GzmK positive cells are clearly elevated (16–18). Numerous GzmK expressing cell types have been identified *in vitro*, with multiple found to secrete GzmK under specific culture conditions (16, 38). Moreover, constitutive secretion of GzmK has been described in a population of CD8^+^ T cells (38). In response to tissue injury and inflammation, it is therefore extremely likely a pool of extracellular GzmK will accumulate. However, due to limitations in the sensitivity of immunohistochemistry, there is an inability to accurately detect extracellular granzymes within these tissues. This makes conclusions about the effect of GzmK accumulation difficult to separate between cellular GzmK or that present extracellularly. There have been recent advances and tools are emerging for the detection of other granzymes in multiple biological samples. Recently, fluorescence-energy resonance-transfer (FRET)-based peptide probes (FAM-peptide-DABCYL) were developed to detect GzmA activity in serum and tissue lysates (42). The development of similar tools for GzmK detection would be enormously useful to elucidate how GzmK accumulates in a range of tissue types. The ability to better understand how GzmK accumulates in disease would inform the development of therapeutic approaches, including inhibitor design.

## 4 Current research gaps: how important is GzmK’s pro-inflammatory role?

**Figure 1:**
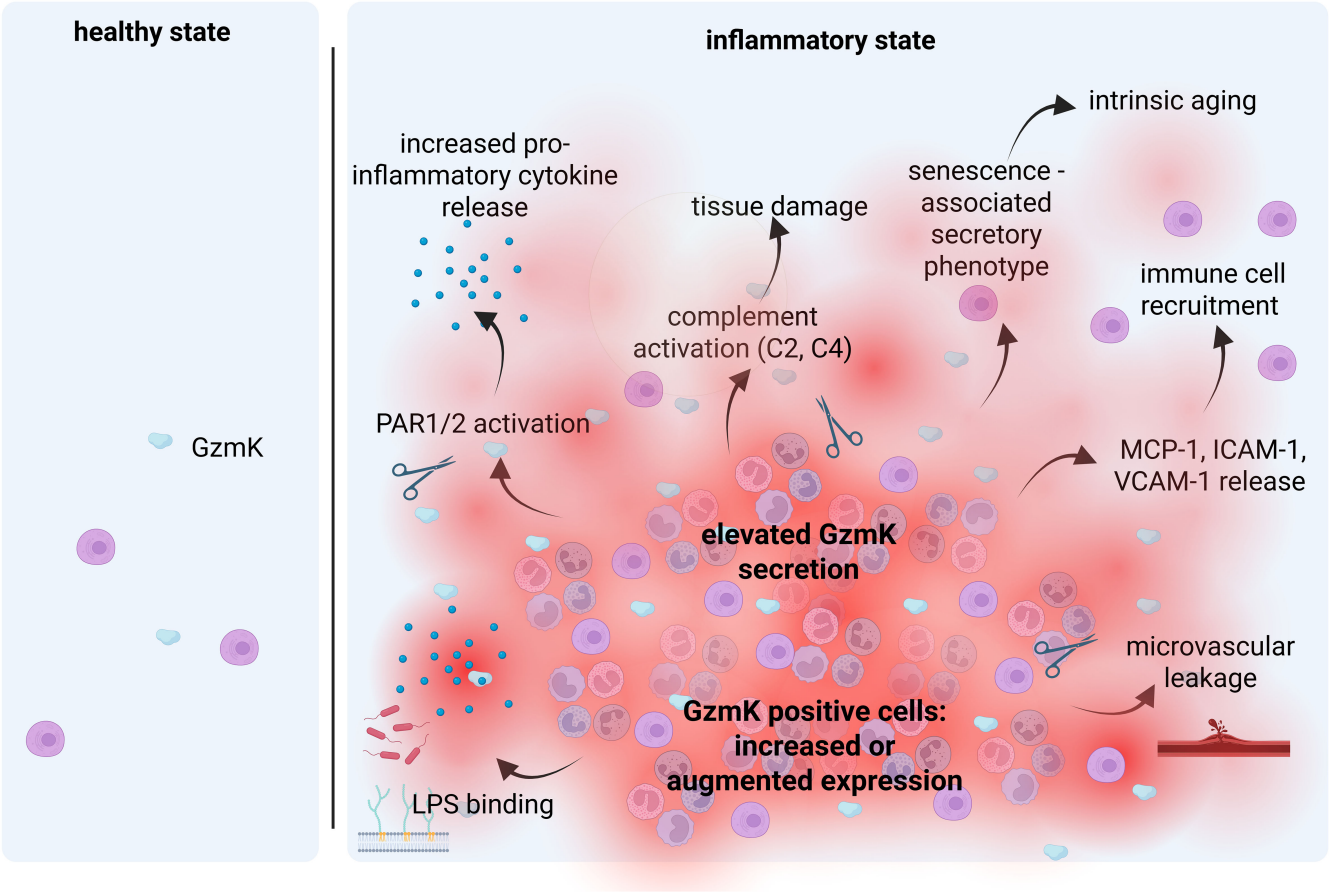
Main mechanisms involved in the GzmK-mediated inflammatory process. There is negligible GzmK in healthy tissues but is elevated in response to tissue injury and inflammation. Enhanced GzmK secretion leads to enhanced immune cell recruitment, elevated pro-inflammatory cytokine detection, complement activation and cell senescence. Reproduced with permission from BioRender.

Emerging evidence over recent years has established GzmK as having pro-inflammatory properties (**Figure 1**). This is, in part, due to its ability to binds to LPS (41), induce pro-inflammatory cytokine expression (18, 40, 41, 43, 44), facilitate immune cell recruitment (44), and activate complement (35). GzmK has also been identified as being a key contributor to inflammaging (19). The most well described mechanisms will be discussed below.

### 4.1 LPS

Human GzmK has been demonstrated to bind to both purified LPS and LPS on Gram-negative bacterial cell walls (41). GzmK modulates toll-like receptor 4 (TLR4) signaling in immune cells, leading to increased pro-inflammatory cytokine expression, including TNF-α from monocytes and IL-1β from macrophages. Together, extracellular GzmK contributes directly to the immune response to bacterial infections. However, based on studies in GzmK^-/-^ mice, the contribution of GzmK to overall disease severity in response to infection appears to be less than other immune-secreted proteases (i.e., GzmA) (29, 30).

### 4.2 Protease-activated receptor

PARs, a subfamily of G protein-coupled receptors, mediate the cellular effects of proteinases. Comprising PAR1, 2, 3 and 4, they have unique but sometimes overlapping roles in inflammation, hemostasis, and thrombosis (45). Multiple studies confirm GzmK to cleave and activate PAR1 (40, 44). This leads to increase pro-inflammatory cytokine secretion and has been observed in multiple cell types, including peritoneal macrophages and cultured M1 macrophages (IL-1β) (16, 46), lung fibroblasts (IL-6, IL-8) (40), keratinocytes and skin fibroblasts (IL-6) (16), and endothelial cells (IL-6) (44). These observations are supported *in vivo*, where GzmK^-/-^ mice with acute burns display decreased IL-1β and IL-6 compared to WT mice (16). GzmK-mediated PAR1 activation in endothelial cells also increases the expression of intercellular adhesion molecule (ICAM)-1, vascular cell adhesion molecule (VCAM)-1, and monocyte chemotactic protein (MCP)-1 (44). This led to increased monocyte attachment to endothelial cells, suggesting GzmK as an immune cell attractant. In support, thermal injured GzmK^-/-^ mice wounds display reduced ICAM-1, VCAM-1, and MCP-1 expression in combination with lower macrophage detection (16).

More recently, GzmK is reported to cleave and activate PAR2 (18, 43). Similarly to PAR1, GzmK-mediated PAR2 activation increases pro-inflammatory cytokine expression (IL-6 and IL-8), which is observed in epithelial cells (43). GzmK activation of PAR2 is separately demonstrated through detection of cleavage on the surface of a reporter cell (nLuc–PAR–eYFP CHO) (18). Mechanistically, GzmK-mediated PAR2 cleavage led to recruitment of β-arrestin and phosphorylation of ERK (43). Notably, both GzmK and trypsin cleave PAR2 at the same location, however, GzmK is unable to induce a classical Ca^2+^ flux.

Multiple naturally expressed proteases have also been described to cleave PAR1 and/or PAR2, including trypsin, plasmin, kallikreins, neutrophil elastase, mast cell tryptase, tissue factor/factor VIIa/factor Xa, activated protein C, and matrix metalloproteinase-1 (47). Some of these proteases may be dysregulated in disease, whilst others are tightly regulated. As an example, although matrix metalloproteinase-1 is elevated in response to tissue injury, it is tightly regulated by tissue inhibitors of metalloproteinases (TIMPs), thereby limiting its ability to act uncontrolled. To better understand the role of GzmK in disease, future studies must therefore elucidate the relative contributions these proteases play in PAR activation and under what conditions does GzmK have the greatest impact. This includes identifying whether GzmK accumulates and increases its proteolytic activity in response to sustained inflammation.

### 4.3 Complement

Lymphocyte-derived GzmK is emerging as having a key role in complement activation (38). GzmK mediates activation through the cleavage of C2 and C4. Ultimately, this results in the generation of C3a, C3b, C4b, and C5a, which are key effectors of complement. This has been observed *in vivo* in rheumatoid arthritis patients, where regions of complement activation correspond to increased GzmK detection (38). Moreover, in arthritis and dermatitis mice, GzmK-mediated complement activation reportedly contributes to disease progression.

Together, it is now clear GzmK mediates a pro-inflammatory phenotype, occurring through multiple and distinct pathways. GzmK will therefore likely have important pathologic roles in multiple disease modalities characterized by inflammation.

## 5 Future developments in the field: will pharmacological inhibition of GzmK reduce disease?

Based on its pro-inflammatory and overall pathogenic effects in multiple disease states, GzmK is emerging as a therapeutic target. Although GzmK inhibitory agents have been described, none are highly specific and with each capable of inhibiting other proteases. IαIp is a naturally occurring physiological inhibitor of GzmK. Found in human and mouse plasma, Plasma IαIp levels are inversely correlated with extracellular GzmK and disease severity in sepsis patients (12). This suggests IαIp to provide a regulatory mechanism (at least in circulation) for limiting the detrimental effects of extracellular GzmK, likely in response to increased GzmK secretion during pro-inflammatory events. IαIp, which also inhibits trypsin, chymotrypsin, plasmin, neutrophil elastase, and cathepsin G (48), has been assessed therapeutically in conditions where there is increased inflammation. Circulating IαIp levels are higher in healthy volunteers than severe sepsis patients (49), thus IαIp delivery was assessed in mice as a potential sepsis treatment. Intravenous IαIp increased survival after an intravenous challenge of *Escherichia coli* (49). In a separate study, intraperitoneal IαIp delivery improved survival to nearly 90% in both LPS induced sepsis and with live bacterial infections (50). IαIp also improved survival after cecal ligation and puncture (51, 52). Intraperitoneal IαIp has additionally been evaluated for anthrax, lacking improved survival outcomes (53). However, combining IαIp and moxifloxacin did improved survival compared to controls including moxifloxacin alone.

The light chain of IαIp, also called bikunin, contains the GzmK inhibitory activity (54), suggesting it may alternatively be used therapeutically. Bikunin is cross-linked in the IαIp complex and requires partial proteolytic degradation to activate. Following cleavage, active bikunin is rapidly cleared from circulation by glomerular filtration and receptor-mediated uptake (55). In rats, intravenous bikunin injection has a half-life of only 10 min. This may account for free bikunin only representing about 2% of total plasma bikunin (reported in (8)). As such, the limited half-life of bikunin may be limiting for therapeutic use unless improved delivery strategies are implemented.

Other non-specific synthetic GzmK inhibitors have been identified, including Phe-Pro-Arg-chloromethyl ketone (PFR-CK), PefablocSC, phenylmethylsulfonyl fluoride, and benzamidine (56, 57). In mice with asthma, PFR-CK, which also inhibits plasma kallikrein, factor XIIa (58) and granzyme A (59), was recently assessed (intraperitoneally every second day), displaying decreased airway eosinophil infiltration, reduced goblet cell hyperplasia, and improved lung function (15). Together, although the number of studies is limited, pharmacological inhibition of GzmK has potential as a therapeutic and warrants further investigation.

## 6 Discussion

It is now clear GzmK has important roles in disease pathogenesis, but many questions remain. More work is required to better understand the relative contributions of different cell types, especially the various CD8^+^ T cell subsets, to the presence of GzmK in diseased tissue. We need better tools to assess extracellular GzmK accumulation in damaged tissues and if elevated, what tissues display the greatest increase. Although GzmK’s role in numerous mechanisms have been described, we need to better uncover GzmK substrates and how increased proteolytic cleavage of these substrates contributes to disease. A greater knowledge of novel substrates will likely lead to the identification of additional mechanisms of action. Remaining a controversial issue, we need to establish the relative contribution of GzmK’s catalytic activity to overall pro-inflammatory mediation. Finally, we need to evaluate the therapeutic potential of pharmacological GzmK inhibition. A better grasp of how GzmK contributes to disease will guide the design of these therapeutics and help select the specific diseases to focus on.
